# Supplemental feeding of yeast cell wall promotes growth of Tibetan sheep by altering rumen fermentation and improving rumen microbiota and liver metabolism

**DOI:** 10.1186/s12866-026-05034-3

**Published:** 2026-05-18

**Authors:** Xiaoxia Han, Ting Liu, Hanfang Zeng, Jianbin Liu, Zengkui Lu, Jianfeng Xu, Jing Wang, Xinhui Yang, Shengxin Zhu, Hang Ran, Lijing An, Faming Pan, Chen Zheng

**Affiliations:** 1 College of Animal Science and Technology, Gansu Agricultural University, Lanzhou, Gansu Province 730070 P. R. China; 2 Lanzhou Institute of Husbandry and Pharmaceutical Sciences, Chinese Academy of Agricultural Sciences, Lanzhou, Gansu Province 730050 P. R. China; 3 Animal Husbandry and Veterinary Station of Nawu Town, Hezuo, Gannan Tibetan Autonomous Prefecture, Gansu Province 747000 P. R. China; 4 Institute of Animal & Pasture Science and Green Agriculture, Gansu Academy of Agricultural Sciences, Lanzhou, Gansu Province 730070 P. R. China

**Keywords:** Yeast cell wall, Liver, Propionic acid, Glycerolipid metabolism, Tibetan sheep

## Abstract

**Background:**

The prolonged cold season on the Qinghai-Tibet Plateau poses substantial challenges for most animals, including limited access to natural pasture, reduced appetite, and subsequent weight loss. The polysaccharides that are contained in yeast cell wall (YCW) act as prebiotics, promoting the action and development of beneficial gut microbiota while inhibiting the proliferation of pathogens, thereby maintaining normal gastrointestinal function in animals.

**Methods:**

This research aimed to examine how incorporating yeast cell wall (YCW) into the diet influences rumen fermentation, the composition of microbiota, and liver metabolism in Tibetan sheep. A total of 30 one-year-old Tibetan sheep, with an mean weight of 30.51 ± 7.07 kg, was randomly assigned to groups: a control group and a supplementation of 0.3% YCW group. Each group consisted of 15 sheep. The experimental period lasted for 98 days.

**Results:**

The research showed that the addition of YCW increased Dry matter digestibility and average daily gain (ADG) of Tibetan sheep significantly (*P* < 0.05); the concentrations of propionic acid, acetic acid, total volatile fatty acids, and ammonia nitrogen in the rumen were significantly increased (*P* < 0.05); In the liver, the mRNA expression of genes associated with gluconeogenesis, including Glucose-6-phosphatase, catalytic subunit (G6PC), Phosphoenolpyruvate carboxykinase 1(PEPCK1), and Fructose-1,6-bisphosphatase (FBP), were significantly increased following YCW supplementation (*P* < 0.05); the mRNA expression level of Sterol regulatory element-binding protein 1c (SREBP1c), which is involved in lipid metabolism, was significantly decreased (*P* < 0.05); The inclusion of YCW in the diet reduced the relative abundance of Desulfobacterota and Firmicutes significantly, and increased *Prevotella*’s abundance significantly in the rumen (*P* < 0.05). Liver metabolites were substantially enriched in the glycerolipid metabolism, glycolysis/gluconeogenesis, and the AMP-activated protein kinase (AMPK) signaling pathway (*P* < 0.05).

**Conclusions:**

YCW supplementation in feed may advance the growth and gain of Tibetan sheep and improve gluconeogenesis. This effect may be ascribed to modifications in the rumen microbiota facilitating propionic acid fermentation, subsequently regulating liver lipid metabolism.

**Graphical Abstract:**

**Figure:**
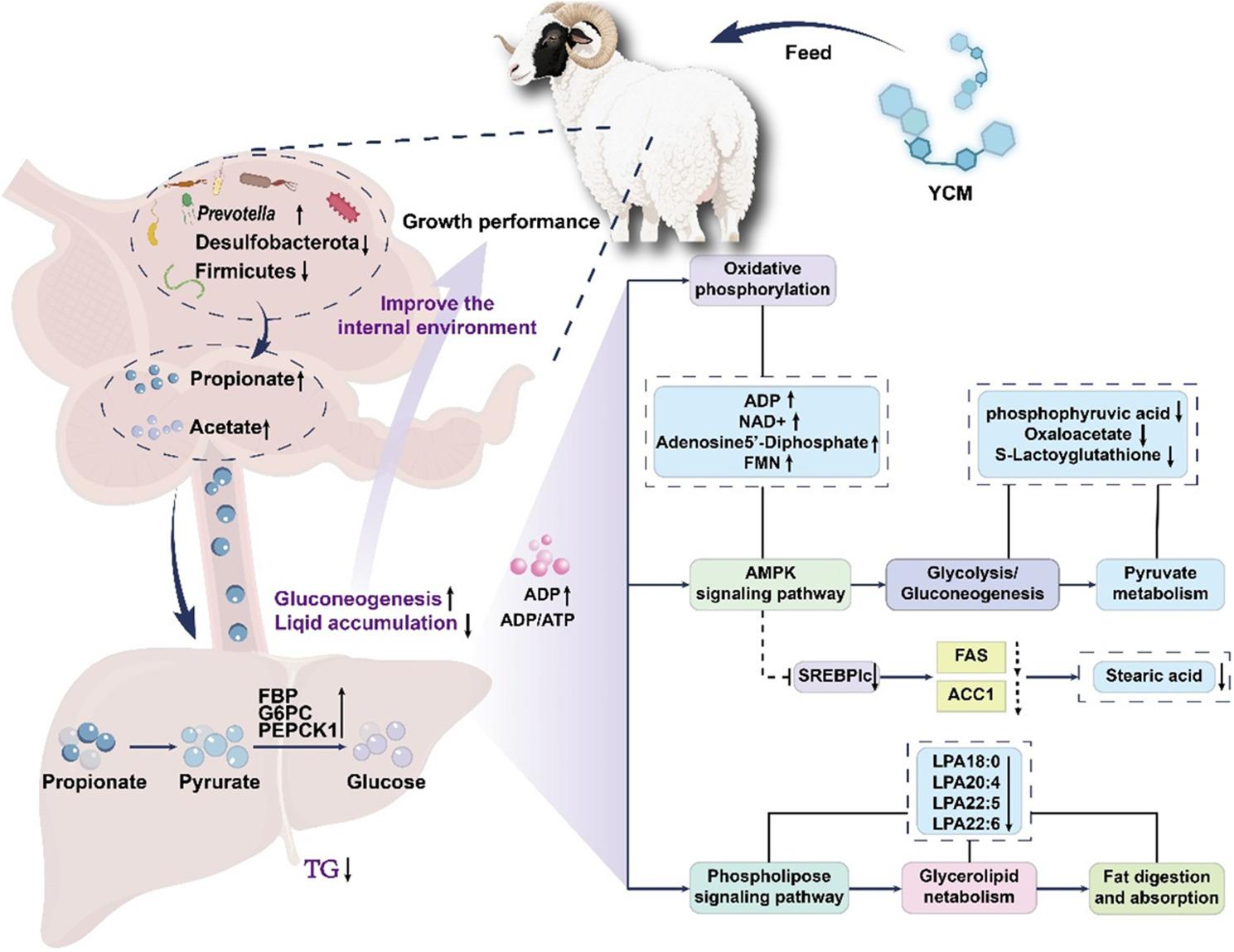
YCW supplements promote propionic acid production by modulating rumen microbial fermentation, thereby enhancing hepatic gluconeogenesis and reducing lipid accumulation.

**Supplementary Information:**

The online version contains supplementary material available at 10.1186/s12866-026-05034-3.

## Introduction

Yeast cell wall (YCW) is a readily available, abundant, and renewable source of yeast polysaccharides [1]. Its primary components include β-1,3 glucan (50%–55% of dry weight), mannan oligosaccharides (35%–40%), β-1,6 glucan (5%–10%), and chitin (1%–2%) [2, 3]. YCW exhibits immune-modulating effects, enhances antioxidant capacity, and mitigates inflammatory responses [4]. Furthermore, beta-glucan serves as a prebiotic stimulating the action and growth of good intestinal flora while inhibiting pathogens’ proliferation. It plays a critical function in the general functioning of the gastrointestinal tract and in the prevention of inflammation [5].

Disruptions in the abundance of microbial communities may increase disease susceptibility and impair host health. Notably, dietary patterns are a key determinant of gut microbiota composition [6]. Studies have demonstrated that supplementing lambs with yeast culture enhances the digestibility of crude protein and cell walls, leading to increased concentrations of rumen propionic acid and plasma glucose [7]. Evidence suggests that pretreatment with a complex dietary polysaccharide derived from yeast can inhibit intestinal inflammation in rats. This intervention shifts the abundance of gut microbiota towards the Bacteroidetes, reduces the abundance of the Firmicutes, and promotes the production of short-chain fatty acids (SCFAs) [8]. In broiler chickens treated with aflatoxin B1 and necrotic enteritis, supplementation with YCW significantly increased the populations of lactobacilli and bifidobacteria in the intestine. This enhancement contributed to a reduction in mortality rates, endotoxin levels, and diamine oxidase concentrations, thereby promoting intestinal health [9].

Overall, YCW can regulate the internal environment of the gastrointestinal tract by altering microbial populations and metabolic processes. In ruminants, carbohydrates in the diet are primarily metabolized by microorganisms to produce SCFAs, with these’s 50–85% being directly absorbed by the rumen epithelium as an energy source for the animals [10, 11]. SCFAs and various other metabolites enter the liver via the portal vein for further utilization [12], forming an ‘axis’ (i.e., the gut-liver axis), that allows interaction between the liver and intestine. Consequently, various gut factors may affect hepatic biochemical metabolic processes and regulate physiological functions [13].

The liver serves as a central hub and a crucial metabolic integrator for energy homeostasis and lipid [14]. Recent research indicates that yeast and its byproducts may improve the metabolic conditions and physiological functions of the liver in various animal species. Specifically, dietary yeast supplements have been shown to reduce lipid droplet content in the livers of lactating HU sheep, upregulate the expression of enzymes associated with lipid β-oxidation (such as PPARα and CPTA), and downregulate the expression of genes related to lipid synthesis (including FAS, PPARγ, and DGA1). Concurrently, these supplements increase the expression of key enzymes involved in gluconeogenesis (such as G6PC and FBP) in the liver, thereby protecting liver health and improving metabolic adaptability through regulating glucose homeostasis and lipid metabolism [15]. Additionally, selenium yeast has been found to protect chicken liver from cadmium-induced necrotic apoptosis by suppressing oxidative pressure and MAPK signaling pathways [16]. Therefore, the supplementation of YCW in animal feed is crucial for regulating liver metabolism and safeguarding liver health.

The Qinghai-Tibet tableland is the highest in the world [17]. Its unique environmental conditions are characterized by low oxygen levels, reduced atmospheric pressure, frigid temperatures, intense ultraviolet radiation, and low humidity. These features pose significant challenges for most animals, particularly during prolonged periods of natural forage scarcity, leading to diminished appetite, stunted growth, and systemic inflammation [18]. Consequently, the metabolic processes in the liver significantly contribute to the growth of Tibetan sheep under hypoxic conditions. Animals in high-altitude settings must modify their metabolic and digestive processes to cope with the challenging conditions presented by low temperatures and reduced oxygen availability. This adaptation is evidenced by increased fat accumulation, decreased blood glucose levels, and elevated triglyceride levels. However, long-term exposure to these conditions can negatively impact the animal’s physiology, through the accumulation of lipids in the liver, which particularly affects liver metabolism adversely [19]. Similarly, studies have indicated that metabolomic measurements reveal that, compared to low-altitude lizards, High-altitude lizards may be better adjusted to making use of carbohydrates as an energy source in preference to lipids. Additionally, high-altitude populations exhibit elevated levels of phospholipids in their livers, likely due to adaptive remodeling of membrane lipids in response to altitude and cold stress [20].

Building upon our research group’s prior trial demonstrating that adding 0.2% mannan oligosaccharides to lamb feed promotes intestinal health [21], and considering the effective addition range of 0.2%–0.5% reported in previous studies, YCW was selected for this investigation at a 0.3% inclusion level. This decision was made as YCW’s primary active components are β-glucan and mannan oligosaccharides. We hypothesize that incorporating yeast cell walls into the basal diet of Tibetan sheep during the cold season on the Qinghai-Tibet Plateau can enhance their growth performance while regulating rumen microbiota and fermentation, improving liver metabolism, and promoting efficient energy utilization. To validate this assumption, we elucidated how the inclusion of yeast cell walls in Tibetan sheep diets promotes growth and health via the gut-liver axis by examining changes in fermentation type and rumen microbiota, alongside changes in hepatic gluconeogenesis and lipid metabolite levels.

## Materials and methods

### Experimental design and animals

The experimental was conducted from September 2023 to January 2024 in Kecaizhen, Xiahe County, Gansu Province, China (mean elevation 3050 m, longitude 102°8′10.35″, latitude 34°51′31.37″). Experimental animals were sourced from the Naorigaqi Ko Sheep Breeding Cooperative. Thirty healthy, 10-month-old male Tibetan rams with identical genetic backgrounds and an average weight of 30.51 ± 7.07 kg were chosen. The sheep were randomly divided into a control group and an experimental group, with the latter receiving a supplementation of 0.3% yeast cell wall in the basal ration. Each group consisted of 15 sheep. Each treatment group comprised five pens/replicates, with three rams per pen/replicate. The yeast cell walls utilized in this experiment were purchased from Angie’s Yeast Co. Ltd (Hubei Province, China). All diets consisted of pellets with a diameter of a length and 3.5 mm ranging from 1 to 2 cm. The formulations and the test feeds’ nutrient compositions are detailed in Table 1. Feeds prepared according to these formulations were administered during an 84-day feeding trial, following a 14-day pre-feeding period. Throughout the trial period, the experimental sheep were housed in pens organized by replicates and fed twice each day (at 09:00 and 17:00), with free access to water. The animals were weighed individually before morning feeding on days 1, 28, 56, and 84 of the trial periods. The average daily gain (ADG) was calculated for each growth stage based on the body weight data and the number of trial days. The daily feed ration offered was recorded. Any remaining feed was collected and weighed before the next morning’s feeding to determine the orts. These data were used to calculate the average daily feed intake (ADFI) and the feed-to-gain ratio (F/G).

**Table 1:** Dietary composition and nutrient content (Dry matter basis, %)

Ingredients	CON	YCW
Corn	46.21	46.21
Soybean meal	7.65	7.65
Safflower meal	3.5	3.5
Corn germ meal	3.0	3.0
Cotton pulp	2.5	2.5
Puffing of soybean	0.82	0.82
Alfalfa hay	7.0	7.0
Corn straw	5.0	5.0
Sunflower hull	5.0	5.0
Soybean hulls	3.0	3.0
Corn husk	10.0	10.0
Salt	0.68	0.68
Molasses	3.0	3.0
CaHPO_4_	1.14	1.14
NaHCO₃	1.0	1.0
Premix^1^	0.5	0.5
Yeast cell wall	0	0.3
Nutrient levels^2^		
Dry matter	90.99	91.50
CP	14.20	14.40
ME, MJ/kg	11.55	11.55
Ca	0.51	0.56
P	0.28	0.25
NDF	32.26	32.68
ADF	13.47	13.49

*Abbreviations*: *CP *crude protein, *ME *metabolizable energy, *NDF *neutral detergent fiber, *ADF *acid detergent fiber

^1^The premix was provided with the following per kg: vitamin A 940 IU, vitamin D 111 IU, vitamin E 20 IU, Fe 25 mg, Mn 40 mg, Zn 40 mg, Cu 8 mg, I 0.3 mg, Se 0.2 mg, Co 0.1 mg

^2^Nutritional levels are actual measured values

### Slaughter procedure

At the end of the feeding trial, six sheep from each group were randomly selected for slaughter and tissue collection underwent a 12-hour period of fasting and water deprivation. Pre-slaughter body weights were subsequently measured and recorded, with slaughter performed via carotid artery bleeding. Following slaughter, rumen segments were promptly excised, rinsed, and fixed in 4% paraformaldehyde to prepare paraffin sections, which were then stained with hematoxylin and eosin for histological and morphological examination. The pH value of the rumen contents was rapidly measured, and the rumen contents’ samples were gathered, flash-frozen in liquid nitrogen, and stored at- 80 °C. These samples were used to assess rumen SCFAs and ammoniacal nitrogen, and to analyze the gastrointestinal microbiota. Furthermore, liver tissues were promptly collected, immersed in liquid nitrogen, and kept at -80 °C for later metabolomic analysis.

### Determination of apparent nutrient digestibility

A seven-day digestion trial was conducted, comprising a three-day pre-trial period and a four-day main trial period. Prior to daily feeding, all faeces excreted by each experimental sheep over the preceding 24 h were collected, weighed, and placed into pre-prepared aluminium containers. Samples were taken at a uniform ratio. Immediately thereafter, the faecal samples were treated with 10% sulphuric acid solution for nitrogen fixation and stored at room temperature for subsequent protein content determination. An additional 300 g fecal sample was weighed and placed in a 65 °C oven for drying, then stored at room temperature for routine nutritional analysis. The calculation methods for relevant digestion indices are as follows [22]:

$$\:\mathrm{A}\mathrm{T}\mathrm{T}\mathrm{D}\left(\mathbf{\%}\right)=\frac{En-Fn}{En}\times\:100$$

In the equation, Fn denotes the specific nutrient content in faeces; En represents the intake of the corresponding nutrient; ATTD signifies the apparent digestibility of the specific nutrient.

### Analytical procedures

#### Histomorphological analysis of the rumen

Tissue samples of intestinal segments fixed in formaldehyde solution are washed overnight with running water. After washing, the samples were dehydrated using a series of ethanol solutions with increasing concentrations, followed by xylene, and then embedded in paraffin [23]. Each tissue’s parts were acquired and stained with hematoxylin-eosin, followed by light microscopy (NIKON ECLIPSE E100) for observation. Photos were taken using a Nikon DS-U3 imaging system, and nipple length and width were measured using C.V. 2.0 software to calculate the corresponding averages.

#### Determination of rumen fermentation parameters

Rumen fluid samples were centrifuged at 12,000×*g* for 10 min at 4°C. One milliliter of supernatant was mixed with 0.2 milliliters of 25% pyrophosphate solution (containing 200 µl of 1% crotonic acid as an internal standard) and incubated at 4 °C for 3 h. After centrifugation, the supernatant is filtered through a 0.22-micron membrane filter and analyzed by gas chromatography to determine short-chain fatty acid concentrations [24].

#### Determination of liver triglyceride and cholesterol concentrations

A 0.5 g sample of liver tissue was homogenized in 4.5 mL of physiological saline (1:9, w/v) at 12,000 rpm for 10 min in an ice-water bath using a mechanical homogenizer. The homogenate was centrifuged, and the resulting supernatant was collected for further analysis. The protein concentration was determined using a microplate assay, and the concentrations of triglycerides and cholesterol were subsequently measured by the same method. The kits employed for these determinations were sourced from Nanjing Jiancheng Bioengineering Institute, China (Catalogue Nos. A045-2, A110-1-1 and A111-1-1), and liver triglyceride and cholesterol concentrations were calculated according to the methods specified in the kits.

#### Real-time fluorescent quantitative PCR of the liver

Total RNA was extracted from approximately 50 mg of liver tissue using 1 mL of TRIzol reagent. First-strand cDNA was synthesized from the extracted RNA using a cDNA synthesis kit (AG11728, Accurate Biology, Nanjing, China) according to the manufacturer’s instructions, with an RNA input of 1 µg. Quantitative real-time PCR (qPCR) was subsequently performed using SYBR Green premix (AG11701, Accurate Biology, Nanjing, China) on the real-time PCR system. Thermal cycling conditions were as follows: 95 °C for 30 s; 95 °C for 5 s, 60 °C for 30 s, repeated for 40 cycles. Relative gene expression levels were calculated using the 2^⁻ΔΔCt^ method, with β-actin as the internal control.Information regarding the primer sequences used for RT-qPCR can be found in Table S1.

#### 16 S rRNA sequencing

A total of 12 rumen fluid samples (6 from the control group and 6 from the YCW group) were subjected to 16 S rRNA gene sequencing. Genomic DNA was extracted from rumen contents using the CTAB method [25]. DNA integrity was assessed by 1% agarose gel electrophoresis, with high-quality samples showing a single intact band without smearing. DNA concentration and purity (A260/A280 ratio) were measured using a NanoDrop spectrophotometer. Samples meeting quality criteria (A260/A280 = 1.8-2.0) were used for library construction. DNA samples were standardized to a concentration of 1 µg/µL. For bacterial DNA amplification, universal primers 338 F (5’-ACT CCT ACG GGA GGC AGC A-3’) and 806R (5’-GGA CTA CHVGG TWT CTAAT-3’) were employed to specifically amplify the V3-V4 region of the 16 S rDNA gene. PCR products were purified using magnetic beads and aliquoted according to concentration. All PCR mixtures were supplemented with 15 µL Phusion ^®^ High-Fidelity PCR Master Mix (New England Biolabs). A total of 0.2 µM primers and 10 ng of genomic DNA template were included in the mixture. Initially, the mixture was denatured at 98 °C for 1 min. This was succeeded by 30 cycles, where the temperature was set at 98 °C for 10 s, then at 50 °C for 30 s, and finally at 72 °C for 30 s. The procedure was finalized with an extension phase at 72 °C lasting 5 min [26]. Amplified products were verified by 2% agarose gel electrophoresis and purified using the Qiagen Gel Extraction Kit. Sequencing libraries were constructed using the TruSeq DNA No-PCR Sample Preparation Kit with indexing, followed by 250 bp paired-end sequencing on the Illumina NovaSeq platform. Raw data were assembled using FLASH software and underwent strict quality control with QIIME to obtain Clean Tags [27]. Clean Tags were aligned against a reference database [28] to remove chimeric sequences, yielding Effective Tags. These were then processed through the DADA2 module in QIIME2 for noise reduction, resulting in amplicon sequence variants (ASVs) [29]. Species annotation was performed using the Mothur method combined with the SILVA138 database [30]. Representative ASV sequences underwent multiple sequence alignment via MUSCLE software to determine phylogenetic relationships. Samples were normalized to identical sequencing depths prior to α and β diversity analyses. Diversity indices (Chao1, Shannon and Simpson) were calculated using QIIME software. PCoA analysis and LDA (threshold 3.5) were performed using R software (version 4.3.0) to identify microbial biomarkers at different taxonomic levels.

#### Metagenomic sequencing

For metagenomic analysis, the same 12 rumen fluid samples (6 per group) were used. Metagenomic DNA library preparation was performed using the NEXTFLEX^®^ Rapid DNA-Seq Library Preparation Kit. DNA was sonicated using the Covaris M220 system to achieve an average length of approximately 350 bp. Subsequently, Illumina-compatible NEXTFLEX adapters were ligated using T4 DNA ligase, followed by purification with AMPure XP magnetic beads to remove unligated free adapters and small DNA fragments (< 150 bp). Purified DNA fragments were amplified in 10 cycles using a high-fidelity PCR master mix. Amplification products were recovered via magnetic beads to obtain target fragments of 350–600 bp, followed by quality control to ensure library concentration ≥ 2 nM. Sequencing was performed on the Illumina NovaSeq™ X Plus platform using paired-end PE150 mode. Diluted libraries were immobilized in flow cells and formed DNA clusters via isothermal bridge amplification. During sequencing, modified DNA polymerase and four-color fluorescently labeled reversible termination dNTPs were sequentially added. Each round extended one base and captured a fluorescent signal, followed by removal of the fluorescent group and termination group to proceed the next cycle, completing 150 cycles. Raw data were converted to FASTQ format and quality-filtered. Data processing involved adapter sequence trimming using fastp software and low-quality read filtering. During processing, host DNA contamination was first removed via the BWA tool, followed by systematic quality assessment of sequencing data using the Fast QC platform. For taxonomic analysis, DIAMOND software (version 2.0.13) performed database alignment on non-redundant genomic amino acid sequences. BLASTP alignment against the NCBI NR database (parameter: e-value threshold 1e^− 5^) was conducted, and species annotation was accurately assigned based on taxonomic data from the NR database [158]. Functional annotation proceeded along two primary dimensions: First, KEGG database alignment (e-value < 1e^− 5^) identified metabolic pathways, enzymatic activities, and modular functions associated with genes. Second, HMMER 3.1b2 software was employed to align sequences against the CAZy database, thereby identifying functional genes such as cellulases and glycoside hydrolases.

#### Untargeted metabolomics analysis

Liver tissue samples from 12 sheep (*n* = 6 per group) were analyzed using LC-MS/MS for untargeted metabolomics. The following protocol was used to process liver samples for untargeted metabolomics analysis. Portions of liver tissue (100 mg each) were pulverized using liquid nitrogen. The resultant tissue homogenate was subsequently resuspended in a pre-chilled solution of 80% methanol and mixed thoroughly using vortex agitation. The samples were subsequently kept on ice for 5 min, followed by centrifugation (4 °C, 15,000 g, 20 min). Obtain a sample of the supernatant and dilute it to reach a final methanol concentration of 53%. The diluted solution was transferred to a fresh test tube, and the centrifugation procedure from the previous step was repeated [31]. The ultimate supernatant was introduced into the LC-MS/MS system for examination. Chromatographic separation was carried out utilizing a ThermoFisher Vanquish UHPLC system.

Data files were analyzed utilizing Compound Discoverer 3.3 (CD3.3, Thermo Fisher), which involved aligning peaks, detecting them, and conducting quantitative assessments for each metabolite. Molecular formulas were estimated by utilizing standardized information. Following this, the peaks were compared to the mz Cloud database (https://www.mzcloud.org/), mz Vault, and Mass List sources to obtain precise qualitative information and relative quantitative measurements. The examination of statistical data was performed utilizing R statistical software (version R-3.4.3), along with Python (version 2.7.6) and the CentOS operating system (version 6.6). This process led to the ultimate identification of metabolites and their corresponding quantification results. After the preprocessing of data and annotation using the Human Metabolome Database and PLS-DA were executed on the gained data. Furthermore, the Kyoto Encyclopedia of Genomes and Genes was applied for metabolic pathways’ analysis.

### Statistical analysis

The initial processing of the experimental data was conducted with Excel (Microsoft, Seattle, Washington, DC, United States). Two-way ANOVA was performed on growth performance using SPSS 26.0 statistical software, with Tukey’s multiple comparison test applied for analysis. Independent samples t-tests were used to analyze slaughter performance, apparent digestibility, rumen fermentation parameters and liver phenotype data. Prior to analysis, the normality of the data was tested using the Shapiro-Wilk test, and homogeneity of variances was tested using Levene’s test, and analyzed through the independent samples t-test using SPSS 22 software. For microbiome and metabolome analyses, the FDR-corrected *P*-value from multiple hypothesis testing yields the q-value. A significance level of *P* < 0.05 was considered statistically significant, while *P* < 0.01 was regarded as highly significant.

## Results

### Effect of YCW supplementation on growth performance

YCW significantly improved the growth performance of Tibetan sheep. The ADG of the YCW group was significantly higher than that of the CON group during the mid-to-late trial period (29–56 days and 57–84 days) (*P* = 0.031, *P* = 0.009), but no significant difference was observed during the early trial period (1–28 days) (*P* > 0.05). Throughout the trial period, the ADFI of Tibetan sheep remained stable, while the feed conversion ratio was significantly lower than that of the CON group in the late trial period (*P* = 0.038), as illustrated in Table 2.

**Table 2:** Effect of YCW supplementation on growth performance of Tibetan sheep (*n* = 5 per group)

Item	Stage/d	CON	YCW	SEM		*P* value	
					Trt	Period	Trt xPeriod
ADFI, kg/d	1–28	1.71	1.70	0.09			
	29–56	1.75	1.73	0.06	0.667	0.907	0.879
	57–84	1.60	1.73	0.06			
ADG, g/d	1–28	247.65	293.99	15.90			
	29–56	162.64	216.56	17.56	0.009	0.007	0.980
	57–84	225.40	258.75	22.61			
F/G	1–28	8.60	7.5	0.60			
	29–56	8.53	8.32	0.91	0.038	0.095	0.426
	57–84	8.15	7.19	1.42			

Period = time effect, Trt x Period = treatment x period interaction

*ADFI* average daily feed intake, *ADG* average daily weight gain,* F/G* feed/gain, *Trt*  treatment effect

### Effect of YCW supplementation on slaughter performance

As shown in Table 3, illustrates that the addition of yeast cell walls did not significantly impact the live weight, carcass weight, or dressing percentage of Tibetan sheep before they were slaughtered.

**Table 3:** Effect of YCW supplementation on slaughter performance of Tibetan sheep (*n* = 6 per group)

Item	CON	YCW	SEM	*P* value
Live weight, /kg	48.13	53.73	1.94	0.160
Carcass weight, /kg	26.20	30.04	1.28	0.141
Dressing percentage, /%	54.44	55.78	0.86	0.465

### Effect of YCW on the apparent digestibility of nutrients in Tibetan sheep

Compared to the CON group, YCW’s addition increased dry matter’s apparent digestibility significantly in the Tibetan sheep diet (*P* < 0.05). However, no significant differences (*P* > 0.05) were noticed between the YCW and CON groups in the apparent digestibility of crude protein, neutral detergent fibre, and acid detergent fibre (Table 4).

**Table 4:** Effect of YCW on the apparent digestibility of nutrients in Tibetan sheep (%)(*n* = 6 per group)

Item	CON	YCW	SEM	*P* value
Dry Matter	62.46	67.02	2.87	0.019
Crude Protein	62.18	64.07	2.20	0.693
Neutral Detergent Fiber	51.98	53.56	2.37	0.761
Acid Detergent Fiber	42.09	43.49	2.15	0.766

### Effect of YCW supplementation on histomorphology and fermentation of the rumen

Supplementary feeding with yeast cell wall did not significantly affect the morphology of rumen papillae or the histomorphometry of the epithelium in Tibetan sheep (Table 5). Histological examination of liver tissue was performed, and representative images are available in Supplementary Figure S1.

**Table 5:** Effect of YCW supplementation to the diet on the rumen histomorphology of Tibetan sheep (*n* = 6 per group)

Item	CON	YCW	SEM	*P* value
Papilla length, /µm	2069.41	2255.90	98.39	0.379
Papilla width, /µm	422.19	449.30	19.41	0.522
Epithelium thickness, /µm	1532.98	1361.37	60.89	0.180

**Fig. 1:**
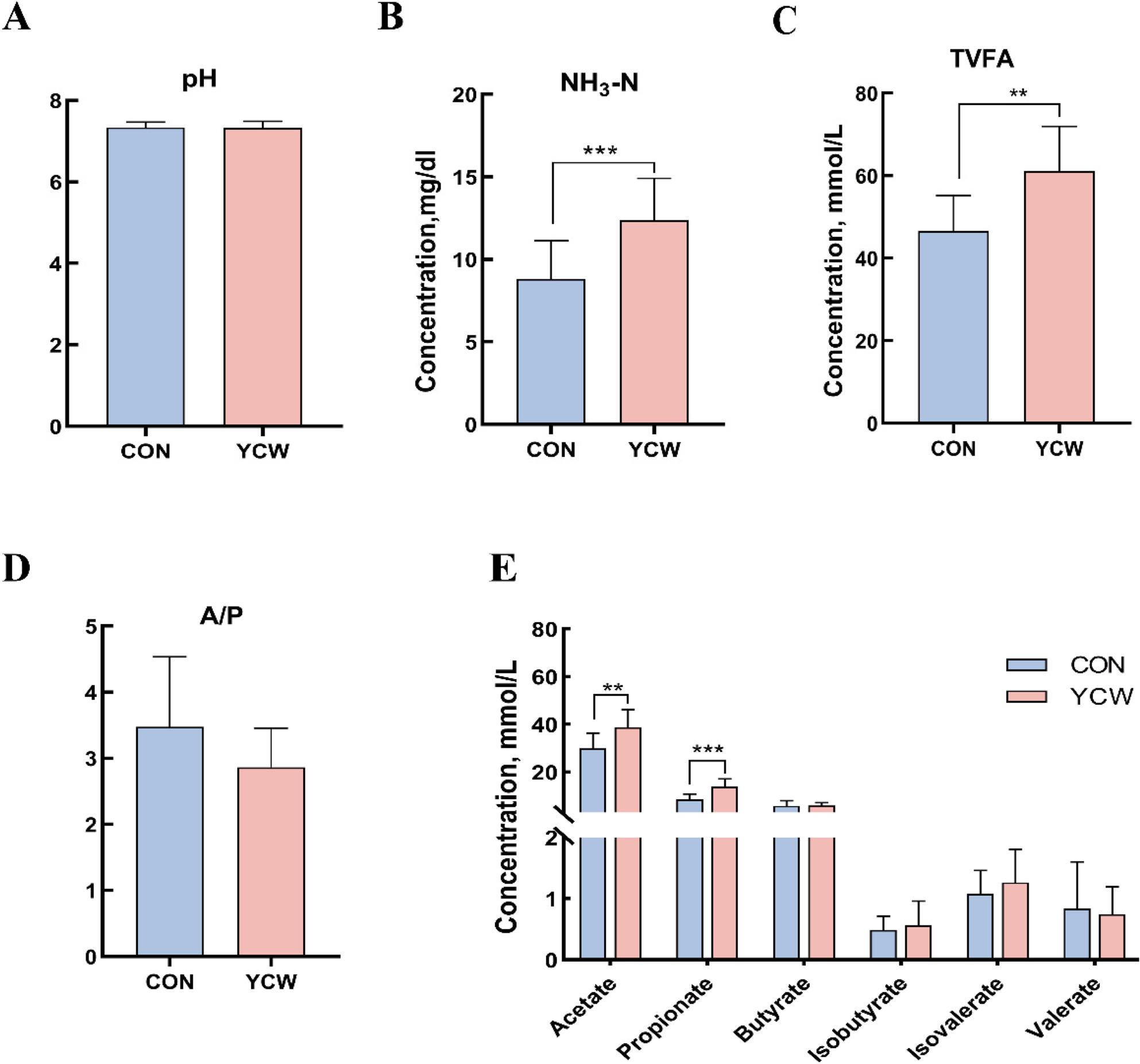
Effect of YCW supplementation on the histomorphology and fermentation of the rumen of Tibetan sheep (n = 6 per group). **A** denote pH. **B **Ammonium nitrogen concentration. **C** Indicates the total concentration of volatile fatty acids. **D** A/P. **E** Indicates the concentration of each volatile fatty acid. ** indicates P < 0.05, *** indicates P < 0.01, CON = control group; YCW = 0.3% Yeast cell walls group; NH3-N= ammoniacal nitrogen; TVFA= total volatile acidity; A/P = the ratio of acetate to propionate concentration

The addition of yeast cell walls does not affect rumen pH (Fig. 1A). The YCW group exhibited a significantly greater concentration of propionate, acetate, TVFA, and NH_3_-N compared to the CON group (Fig. 1B-E) (*P* < 0.05).

### Effect of YCW supplementation on rumen microbiota

#### Rumen microbiota difference using 16 S rRNA gene sequencing

The microbiota within the rumen contents of lambs was comprehensively analyzed utilizing 16 S rDNA sequencing. About 1,274,948 reads were acquired, resulting in 983,110 effective tags after splicing and filtering for subsequent analyses. Each sample generated at least 74,555 effective tags (Table S2).

**Fig. 2:**
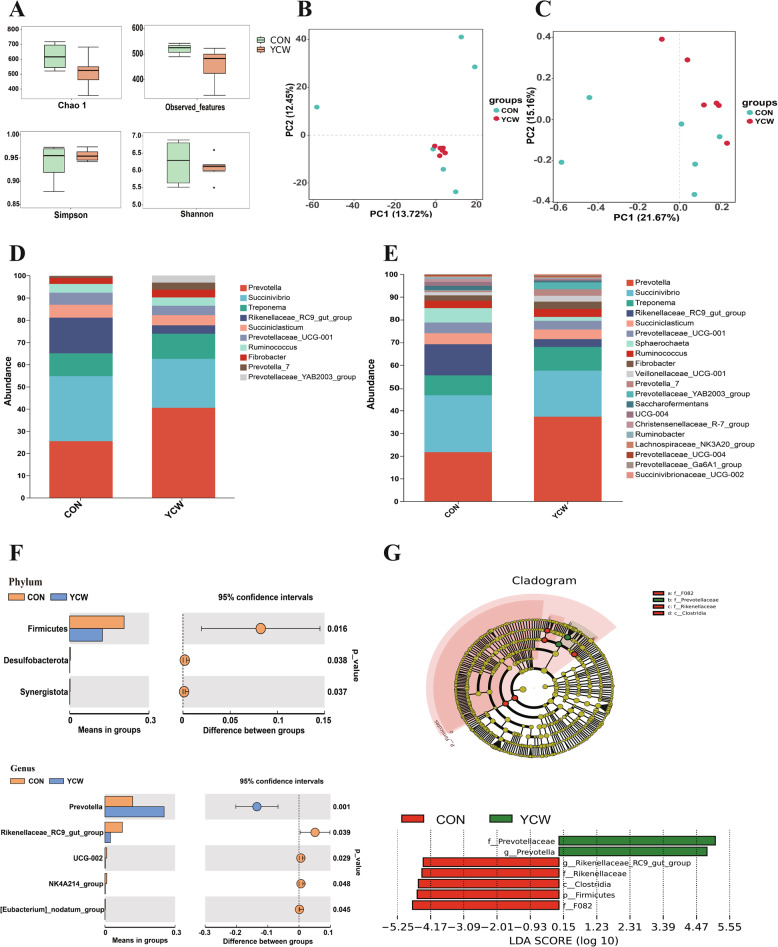
Differences in bacterial community diversity, abundance, and structure in the rumen of Tibetan sheep after feeding yeast cell wall (n = 6 per group). **A** Community’s diversity and richness. **B** PCA analysis. **C** OPLS-DA analysis. **D** and **E**. Microbial composition at the phylum and genus level **F**. T-test for microbiological differences **G**. Histogram and cladogram of LDA value distribution between groups

The α diversity index did not exhibit significant changes (Fig. 2A). The results of principal component analysis (Fig. 2B) and principal coordinate analysis (Fig. 2C) showed that, A significant level of differentiation was observed between the rumen microbial communities of the two groups after the addition of yeast cells. In terms of microbial community composition, the dominant phylum were Firmicutes (16.50%) and Bacteroidota (57.31%) (Fig. 2D). The predominant genera included *Prevotella* (18.59%), *Succinivibrio* (13.73%), and *Rikenellaceae_RC9_gut_group* (8.66%) (Fig. 2E). The t-test revealed that, At the phylum level, the control group’s rumen exhibited a significantly greater relative abundance of Firmicutes compared to the YCW group, while Desulfobacterota and Synergistota were significantly lower in the YCW group (*P* < 0.05). At the genus classification level, there was a notable rise in the relative abundance of *Prevotella* in the rumen following the administration of yeast cell wall, alongside *Howardella*, while *Rikenellaceae_RC9_gut_group*, *UCG-002*, *Lachnospiraceae_UCG-010*, *NK4A214_group*, *UCG-009*, *[Eubacterium]_nodatum_group*, and *Marvinbryantia* were significantly lower (*P* < 0.05) (Fig. 2F).Analysis of the lefse indicated that the YCW group’s rumen tissue was enriched with biomarkers such as Prevotellaceae and *Prevotella*, whereas those in the control group comprised *Rikenellaceae_RC9_gut_group*, Rikenellaceae, Clostridia, Firmicutes, and F082 (*P* < 0.05) (Fig. 2G).

#### Differential rumen microbiota and their taxonomic indicators revealed through metagenomic sequencing

**Fig. 3:**
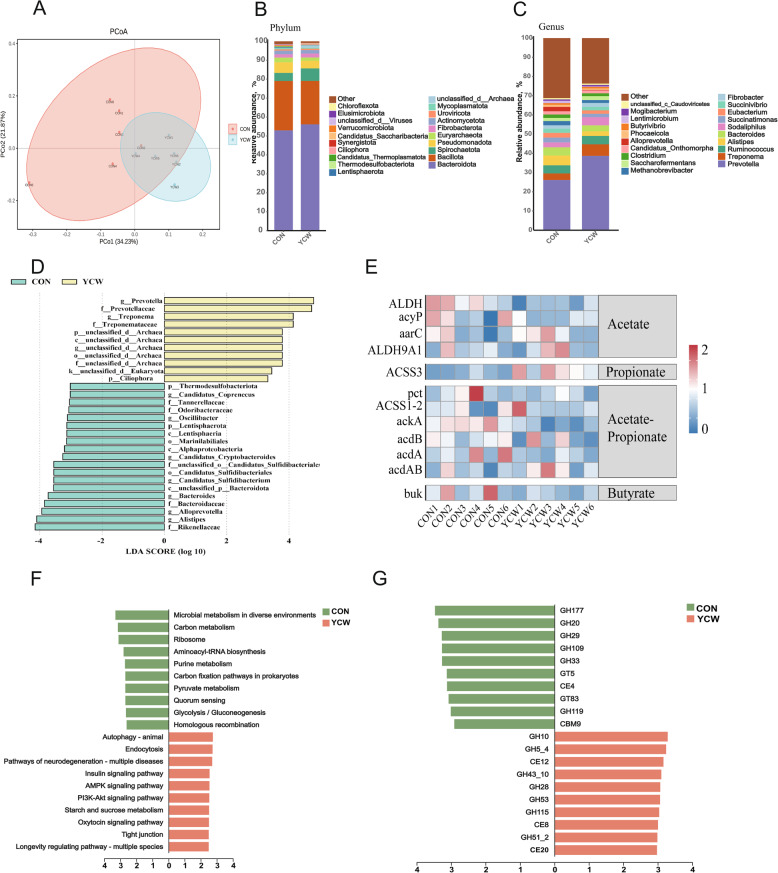
Faecal microbiota of CON and YCW Tibetan sheep can be distinguished using metagenome sequencing(n = 6 per group). **A** The PcoA analysis of CON and YCW Tibetan sheep. **B** and **C** The relative abundance of microbial composition at the phylum level and genu level. **D** LDA score plot of enriched archaea taxa abundance determined by linear discriminant analysis effect size (LEfSe) analysis (LDA value > 3.5; P-value < 0.05. **E** Heatmap of expression abundance for selected genes associated with SCFA production. **F** Differential Kegg functions based on pathway level 3. **G **Differential CAZyme functions based on family-level enzymes (Wilcoxon rank-sum test, P < 0.05)

Metagenomic sequencing results indicate that the alpha diversity indices (Ace and Chao indices) of the rumen microbial community showed no significant differences. However, the Shannon index was significantly reduced, while the Simpson index significantly increased in the YCW group (*P* < 0.05) (Fig. S1A). Species composition analysis revealed Bacteroidetes and Bacillota as the dominant phyla, with *Prevotella*, *Alistipes*, *Ruminococcus*, *Treponema*, and *Bacteroides* ranking highest in relative abundance. Notably, the abundance of *Prevotella* increased in the YCW group, which is consistent with the findings from 16 S rRNA gene sequencing (Fig. 3B-C). Lefse analysis revealed significant enrichment of the genera *Prevotella*,* Treponema*, and *unclassified_d__Archaea* in the YCW group, while the genera *Candidatus_Cryptobacteroides*, *Bacteroides*, and *Alloprevotella* were significantly enriched in the CON group (*P* < 0.05) (Fig. 3D). We specifically analyzed the biosynthetic pathways of SCFAs and the genes encoding enzymes associated with SCFA biosynthesis. Among these, the acetate-related genes *aarC* and *ALDH9A1*, as well as the propionate-related gene *ACSS3*, exhibited higher relative abundances in the YCW group. Genes *acdA*, *acdB*, and *acdAB*, which are associated with both acetate and propionate, were distributed across both groups. Conversely, the butyrate-related buk gene showed a higher abundance in the control group (Fig. 3E).

Regarding functional annotation, the PcoA revealed distinct differences between the two groups concerning the functional annotations from the KEGG and CAZY databases (Fig. S1). Pathway level 3 KEGG gene functions were predominantly enriched in metabolic pathways and biosynthesis of secondary metabolites. Within the CAZY database, enrichment was primarily observed in the GT2_Glycos_transf_2, GT4, CE1, and GT41 families (Fig. S2). Functional pathways’ Further Lefse analysis revealed that the CON group showed significant enrichment in pathways including Glycolysis/Gluconeogenesis and Pyruvate metabolism, whereas the YCW group exhibited significant enrichment in Starch and sucrose metabolism, the PI3K-Akt signalling pathway, and the AMPK signalling pathway; Within the CAZY database, the CON group exhibited higher abundances of GH177, GH20, GH29, GH109, GH33, GT5, CE4, GT83, and CBM9; the YCW group showed higher abundances of GH10, GH5_4, CE12, GH43_10, GH28, GH53, GH115, CE8, GH51_2, and CE20(*P* < 0.05) (Fig. 3F-G).

### Effect of YCW supplementation on liver metabolomics

**Fig. 4:**
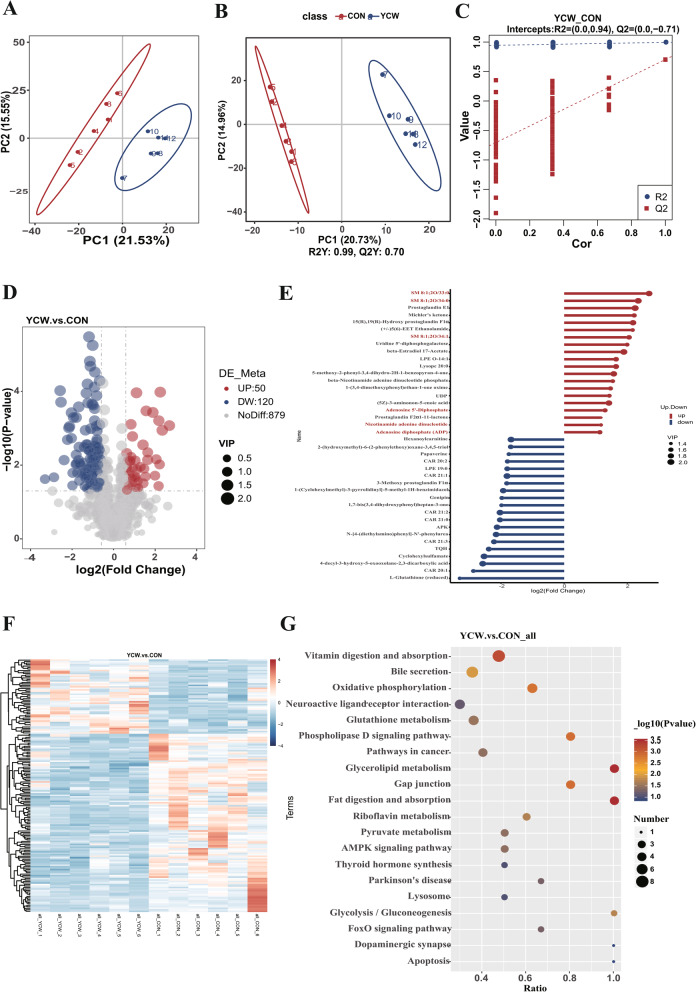
Effects of YCW supplementation on the metabolome of Tibetan sheep liver(n = 6 per group). **A** and **B** The results of PCA and PCoA analysis, respectively. **C** A comparison of the PLS-DA ranking test diagram. **D** A volcano plot of all metabolites, with red and blue patterns representing up-regulated and down-regulated metabolites in the 0.3% YCW group, respectively. **E** The sample comparison matchstick plot. **F** The heatmap of total differential metabolites. **G** The KEGG pathway enrichment analysis

The analyses conducted through PCA and PCoA distinctly revealed notable discrepancies in metabolites across the two groups (Fig. 4A-C). This finding indicates that feeding yeast cell wall polysaccharides can effectively influence liver metabolite changes in Tibetan sheep. A total of 1,049 liver metabolites were discovered in Tibetan sheep (Table S2). Differential metabolites were analyzed using the criteria of VIP > 1.0, FC > 1.5, or FC < 0.667 with *P* < 0.05 (Fig. 4D). In total, 170 metabolites showing differences were identified, which included 50 that exhibited up-regulation and 120 that showed down-regulation (Table S2).

Among the differentially expressed metabolites identified, the top 20 significantly upregulated metabolites include SM 8:1;2O/33:0, SM 8:1;2O/34:0, SM 8:1;2O/34:1, ADP, and NAD+ (Fig. 4E). The relative concent rations of glycerophospholipids metabolites were downregulated in the YCW group, with the exception of Lysopc 20:0. Most differential metabolites classified as fatty Acyls showed downregulation, including Hexanoylcarnitine, Palmitoylcarnitine, Propionylcarnitine, and Acetyl-L-carnitine. Furthermore, there was a downregulation of Stearic acid, Thromboxane B2, and 6β-Prostaglandin I1, whereas Prostaglandin E1 and 15(R),19(R)-Hydroxy prostaglandin F1α exhibited an upregulation. Additionally, most differential metabolites categorized as carboxylic acids and their derivatives showed upregulation. Additionally, two differential metabolites classified as indoles and their derivatives, namely, indole-3-lactic acid and JWH 018 N- pentanoic acid metabolite, were identified as up-regulated in the YCW group. The cluster heat map demonstrates notable variations in the relative concentrations of metabolites observed between the two groups (Fig. 4F). Subsequent KEGG functional annotation and enrichment analysis indicated significant variations in the metabolites that showed differential expression in the livers of Tibetan sheep under diverse conditions. These differentially expressed metabolites were notably enriched in pathways related to Glycerolipid metabolism, Fat digestion and absorption, Vitamin digestion and absorption, the Phospholipase D signaling pathway, oxidative phosphorylation, Bile secretion, Glycolysis/Gluconeogenesis, the AMPK signaling pathway, and Pyruvate metabolism (*P* < 0.05) (Fig. 4G). Among the differential metabolites identified, LPC 18:3, LPA 20:4, LPA 18:0, and LPA 22:6were associated with several pathways were connected with lipid metabolism, including Glycerophospholipid metabolism, Fat digestion and absorption, and Glycerolipid metabolism. Notably, all these metabolites exhibited significant down-regulation in the YCW group (*P* < 0.05). Furthermore, Glycerophospholipid metabolism was associated with PE 36:4, PC 40:5, LPC 22:6, and Acetylcholine, all of which were also significantly down-regulated in the YCW group (*P* < 0.05). Additionally, Phosphopyruvic acid and Oxaloacetate were linked to Glycolysis/Gluconeogenesis and demonstrated significant down-regulation in the YCW group (*P* < 0.05) (Table S2).

### Effects of YCW supplementation on hepatic triglyceride、cholesterol levels, and the expression levels of genes related to glucose and lipid metabolism

**Fig. 5:**
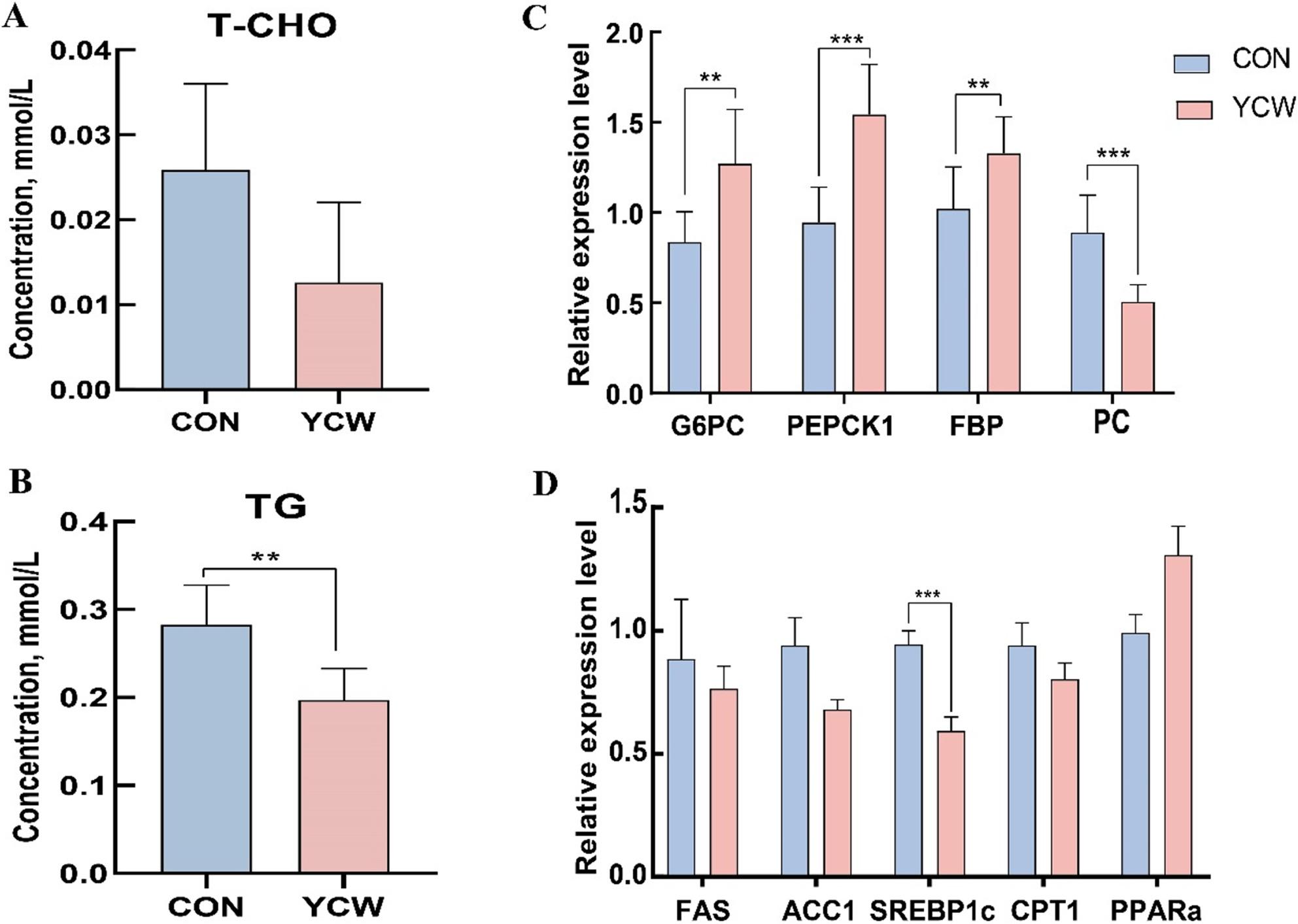
Effects of YCW supplementation on the expression of genes associated with glucose metabolism and lipid metabolism in the liver of Tibetan sheep (n = 6 per group). **A** Triglyceride concentration. **B** Cholesterol concentration. **C** gluconeogenesis-related genes. **D** lipid metabolism-related genes. **P < 0.05, *** P < 0.01

Supplementation with YCW significantly reduced triglyceride concentrations in Tibetan sheep livers (*P* < 0.05) (Fig. 5A), while cholesterol concentrations showed no significant difference between the two groups (Fig. 5B). Given that differentially presented metabolites were significantly enriched in pathways like lipid metabolism, glycolysis/gluconeogenesis, and AMPK signaling, we further analyzed central genes’ expression within these pathways. The levels of mRNA expression for genes associated with gluconeogenesis in the liver, including G6PC, PEPCK1, and FBP, were markedly increased in the YCW group, while the mRNA expression level of PC indicated a significant reduction (*P* < 0.05) (Fig. 5C). Additionally, the mRNA expression levels of SREBP1c, a transcription factor implicated in lipid metabolism, were significantly reduced in the YCW group (*P* < 0.05). Nonetheless, the expression levels of ACC1, FAS, CPT1, and PPARα did not show any notable differences when comparing the two groups (Fig. 5D).

### Correlation between rumen microbiota, fermentation parameters, gluconeogenesis, lipid metabolism-related genes, and metabolites

**Fig. 6:**
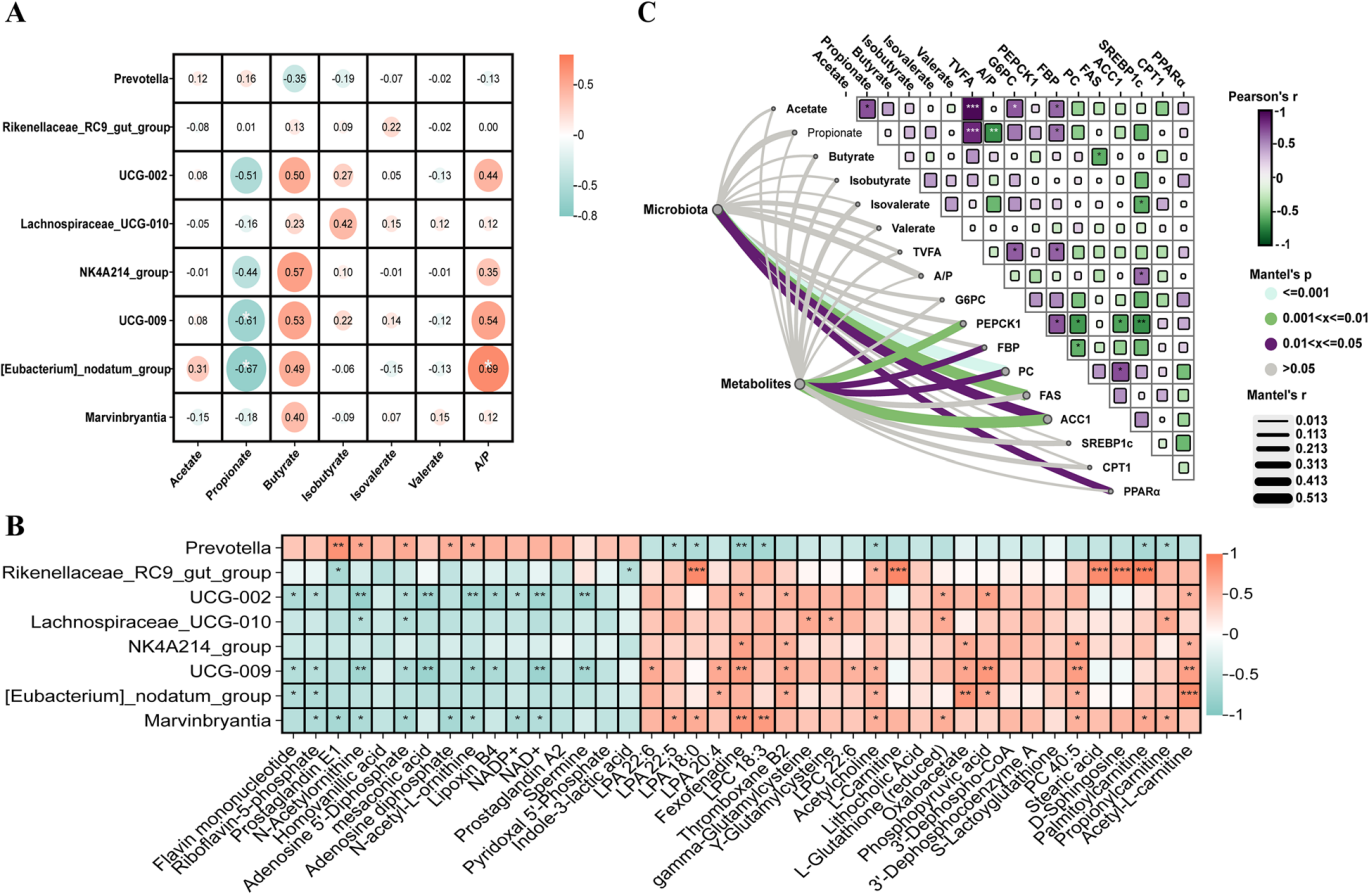
Statistical Spearman correlation between rumen microbial genera, liver metabolites, fermentation parameters, and genes related to gluconeogenesis and lipid metabolism(n = 6 per group). **A** Spearman correlation analysis assessed the association between microb ial genera and fermentation parameters. **B** The analysis of the network heatmap using the Mantel test showed connections among microbial genera, metabolites, fermentation parameters, and genes associated with gluconeogenesis and lipid metabolism. **C** Spearman correlation analysis examined the relationship between microbial genera and liver metabolites (primarily enriched in glycerophospholipid metabolism, gluconeogenesis, and AMPK signalling pathways). *P < 0.05, **P < 0.01, ***P < 0.001

The possible link between gut microbiota, fermentation factors, and metabolites is examined using Spearman correlation analysis. The findings suggest that there is a beneficial correlation between the levels of *Prevotella* and the amounts of acetate and propionate present. Conversely, the relative abundance of U*CG-002*, *Lachnospiraceae_UCG-010*, *NK4A214_group*, *UCG-009*, and *[Eubacterium]_nodatum_group* are negatively correlated with propionate levels, while exhibiting a positive correlation with butyric acid concentrations (Fig. 6A). Furthermore, strong correlations were observed through Mantel test analysis (Fig. 6B) between rumen microbiota and liver metabolites, rumen fermentation parameters, as well as genes connected to hepatic gluconeogenesis and lipid metabolism. Specifically, rumen microbiota showed strong correlations with expression of PC, FAS, ACC1, and PPARα; simultaneously, Metabolites from the liver showed significant associations with expression of PEPCK1, FBP, PC, FAS, and ACC1.

Furthermore, propionate demonstrated a positive correlation with G6PC, PEPCK1, and FBP, whereas it exhibited a strong negative correlation with lipid synthesis-related genes, including SREBP1c and ACC1. Moreover, *Prevotella* is positively correlated with flavin mononucleotide, riboflavin-5-phosphate, prostaglandin E1, adenosine diphosphate, NAD+, NADH, and indole-3-lactic acid. In contrast, it is negatively correlated with glycerophospholipid metabolites, lithocholic acid, oxaloacetate, phosphopyruvic acid, 3’-dephospho-CoA, 3’-dephosphocoenzyme A, stearic acid, and others. Notably, the relative abundance of other microorganisms showed the opposite relationship with these metabolites (Fig. 6C).

## Discussion

This research discovered that the addition of YCW to the rations of Tibetan sheep resulted in increased ADG and a decrease in the F/G. Previous research has demonstrated that supplementation with 0.025% and 0.01% YCW can enhance the whole weight gain and final body weight in broiler chickens [32]. The apparent digestibility of dietary nutrients constitutes one of the key determinants of animal growth performance, while also directly reflecting digestive capacity. The results of this study indicate that the YCW group significantly enhanced the apparent digestibility of DM in the Tibetan sheep diet. The enhancement of dry matter digestibility might be linked to the particular regulatory influence that yeast cell walls have on the fermentation processes of rumen microbes. Although the apparent digestibility of ADF and NDF in the YCW group of Tibetan sheep was higher than that in the CON group, the difference was not significant. It shows indicates that YCW has the potential to promote fibre degradation, However, Extra researches might be needed to fully understand its role in the regulation of fiber digestion. The incorporation of yeast cell walls also enhances rumen fermentation, primarily by increasing the concentrations of acetic and propionic acids while reducing the A/P [33]. The A/P serves as an indicator of an animal’s feed energy utilization efficiency; A reduction in this ratio indicates enhanced efficiency in energy utilization from ruminant feed [34], which aligns with the observed enhancement in growth performance of Tibetan sheep receiving YCW supplements. The proportions of VFAs produced are largely influenced by the animal’s diet [35]. This suggests that YCW supplementation modifies the composition of ruminal fermentation substrates. The increase in NH_3_-N levels is recognized as the main nitrogen source for the synthesis of microbial protein in the rumen, which is accompanied by an upsurge in microbial protein production [36]. The incorporation of yeast cell wall feed enhanced protein degradation in Tibetan sheep, which may contribute to the observed increase in rumen NH3-N concentration. Consequently, the increased concentrations of acetate, propionate, and total volatile fatty acids in the rumen of Tibetan sheep following supplementation with YCW are encouraging indicators of improved energy utilization efficiency and a reduction in cold stress.

In this study, YCW supplementation significantly reduced the relative abundance of Firmicutes, Desulfobacterota, and Synergistota. However, the relative abundance of *Prevotella* significantly increased. The Clostridia within Firmicutes plays a dual role in rumen ecology: it comprises both functional microflora involved in fiber degradation [37, 38] and groups with potential for protein putrefaction. Over proliferation of these groups may lead to the accumulation of metabolites such as ammonia, amines, and hydrogen sulfide, disrupting gut ecological balance and adversely affecting host health [39]. The YCW-mediated downregulation of Clostridia abundance may help regulate these bacteria to a more suitable ecological niche level.

This approach preserves their beneficial functions while reducing the potential threat of their metabolic activities to gut health. Synergistota was found to be positively correlated with NH3eN concentration which increases the absorption and utilization of nutrients [40]. Desulfobacterota, being a Gram-negative bacteria, can produce endotoxin such as LPS, which in turn induces an inflammatory response in the body [41]. The genera within the *Prevotellacea* family typically possess a diverse array of polysaccharide-utilizing motifs and carbohydrate-active enzymes, which may facilitate VFA fermentation in the gastrointestinal tract [42, 43]. Notably, *Prevotellaceae UCG-001* has been shown to produce higher levels of propionate [44, 45]. Furthermore, one study found that *Prevotellaceae_UCG-001* was significantly downregulated in the feces of mice subjected to both a high-fat diet and high fructose intake, and this reduction was reversed by the administration of various plant polysaccharides [46, 47]. The inclusion of yeast cell walls in this study was associated with both an increased abundance of *Prevotella* and elevated propionic acid content, suggesting a potential role for this genus in the observed changes in fermentation patterns. The enrichment of GH5_4 (cellulase), GH10 (xylanase), and GH43_10 suggests that YCW improves the microbiota’s capacity to degrade plant fibers (cellulose, xylan, and pectin). These enzymes break down oligosaccharides to produce SCFAs [48]. Carbohydrate esterases (CE) further support this process by cleaving side chains from modified xylose units [49]. The abundance of relevant genes in the YCW group was significantly upregulated. This improvement enhances the rumen microbiota’s capacity to degrade fibrous feed. Therefore, supplementing with YCW can optimize the abundance of functional bacteria within the rumen, thereby enhancing feed digestibility and increasing short-chain fatty acid levels, which improves the growth performance of Tibetan sheep. Meanwhile, the reduced attachment capacity of these potentially harmful microorganisms helps alleviate epithelial cell inflammation and promotes rumen health.

The liver is commonly acknowledged as the primary organ responsible for the synthesis of fats, the production of glucose from non-carbohydrate sources, and the metabolism of cholesterol [50]. In this study, we observed that several metabolites associated with lipids, particularly phospholipids, were predominantly down-regulated in the YCW group, including LPA, LPC, and LPS. LPC and LPA are crucial in glycerophospholipid metabolism, serving as intermediates, signaling molecules, and modulators of enzyme activity [51, 52]. Furthermore, LPC is involved in regulating host energy [53], while LPA metabolism modifies cellular differentiation to promote oxygen and nutrient supply [54]. Phosphatidylcholine (PC) and phosphatidylethanolamine (PE) being the two primary Glycerophospholipids [55]. In this study, the differential metabolites PE 36:4 and PC 40:5 were significantly down-regulated following the supplemental feeding of YCW. The content of various phospholipid molecules, including sphingomyelin (SM) 8:1;2O/33:0, SM 8:1;2O/34:0, and SM 8:1;2O/34:1, increased following YCW supplementation. Sphingomyelin exhibits strong structural stability [56]. These metabolites that showed differential expression were mainly concentrated related to glycerophospholipid activity and lipid metabolism. The AMPK signaling pathway, crucial for lipid metabolism’s regulation, is abundant in three specific metabolites: adenosine diphosphate (ADP), nicotinamide adenine dinucleotide (NAD+), and adenosine 5’-diphosphate. In eukaryotic cells, AMPK is integral to the maintenance of fatty acid metabolism and the regulation of cellular energy homeostasis. In response to variations in intracellular adenine nucleotide concentrations, AMPK is triggered primarily by a rise in the AMP/ADP ratio in comparison to ATP levels [57, 58]. This study found that the mRNA expression of SREBP1c, a transcription factor related to lipid synthesis and regulated by the AMPK signaling pathway, was reduced in the YCW group. Although ACC1, FAS, CPT1, and PPARαmRNA levels remained unchanged, hepatic triglyceride (TG) content decreased significantly following YCW supplementation. SREBP-1c serves as a principal regulator of gene expression involved in lipogenesis and hepatic triglyceride synthesis [59]. SREBP-1c controls not only ACC1 and FAS, but also other key enzymes involved in TG assembly, such as GPAT and DGAT [60, 61]. Reduced SREBP-1c expression may therefore suppress TG synthesis through these unmeasured genes, even if ACC1 and FAS are unaffected. Additionally, ACC1 activity is regulated post-translationally—its product malonyl-CoA inhibits CPT1-mediated fatty acid oxidation [62]. Thus, the observed TG reduction likely results from combined effects on both lipid synthesis and oxidation. This requires further research. Moreover, earlier research has suggested that SCFAs modulate the AMPK activity of AMPK. Acetic acid enhances the AMP to ATP ratio and activates AMPK, whereas propionic acid diminishes lipid synthesis in hepatocytes by activating AMPK [63]. Consequently, supplementation with YCW may directly modulate liver lipid metabolism genes through the SCFA-AMPK pathway.

Additionally, it may alter the AMP/ADP ratio by increasing the levels of the differential metabolite ADP, which subsequently influences the AMPK signaling pathway to regulate lipid metabolism and energy balance in the liver of Tibetan sheep.

The liver is crucial for energy metabolism and helps in maintaining blood glucose balance because of its role in regulating gluconeogenic processes [64]. This study found that after supplementary feeding with YCW, the mRNA expression levels of G6PC, PEPCK, and FBP genes associated with gluconeogenesis increased in the liver, further confirming enhanced gluconeogenesis. In hypoxic environments, animals increasingly depend on glycolysis for ATP production; glucose is transformed into pyruvate rather than proceeding into the tricarboxylic acid (TCA) cycle, and pyruvate is metabolized into different end-products to regenerate NAD+ [65]. However, in this study, NAD+ levels were elevated in the YCW group. Ensuring a constant supply of glucose in the liver, which usually comes from glycogen reserves, is crucial for the survival of the whole organism under hypoxic conditions. However, the present study found that phosphopyruvic acid and oxaloacetate were downregulated in the liver following supplemental feeding of yeast cell walls. This observation is consistent with the reduction in mRNA levels of the PC gene. The increased propionic acid content observed following YCW supplementation provides a direct mechanistic link to the enhanced hepatic gluconeogenic capacity evidenced by upregulation of PEPCK, FBPase, and G6Pase. Under enhanced flux driven by upregulated PEPCK, FBPase, and G6Pase, the steady-state levels of oxaloacetate and phosphoenolpyruvate would be rapidly consumed [66], explaining their observed decrease. Propionic acid is the most significant substrate in gluconeogenesis, accounting for over 50% to 60% of substrates utilized in gluconeogenesis in ruminants [67]. Importantly, propionate enters gluconeogenesis at the level of succinyl-CoA, bypassing the pyruvate carboxylase (PC)-catalyzed step entirely [68]. This explains the concurrent downregulation of PC observed in this study: when propionate supply is abundant, the liver preferentially utilizes this substrate while reducing carbon entry from pyruvate-dependent pathways, consistent with reports that propionate suppresses gluconeogenesis from amino acids [69]. However, the specific mechanism of action requires further investigation. In summary, the supplementation of yeast cell walls increases the concentration of propionic acid, which serves as an energy source in the rumen of Tibetan sheep, and modifies the pathways that regulate energy metabolism in these animals. Therefore, the incorporation of yeast cell walls enhances energy utilization efficiency. Although the findings after supplementing Tibetan sheep with yeast cell walls were obtained under controlled conditions with consistent background parameters across groups, future studies should validate these results within diverse grazing systems.

## Conclusion

This study demonstrates that dietary yeast cell wall supplementation is associated with enhanced rumen fermentation potential and shifts in microbial community composition, concurrent with improvements in hepatic lipid metabolism, reduced triglyceride accumulation, and upregulated gluconeogenesis in Tibetan sheep. These changes may facilitate more efficient energy utilization, potentially contributing to reduced body weight fluctuations and improved health maintenance.

## Supplementary Information

Supplementary Material 1. Table S1. Primer applied for real-time qPCR measurement. Table S2. 16 S rDNA sequencing reads; Total of metabolites and differential metabolites in liver; KEGG pathways of metabolite of liver. FigureS1. Histological analysis of rumen tissue. Figure S2. The Rumen microbiota of CON and YCW Tibetan sheep using metagenome sequencing.

Supplementary Material 2. P-values after adjustments (microbiome and metabolome).

## Data Availability

The data analysed in this study may be obtained from the corresponding author upon reasonable request. 16 S rRNA sequencing data and metagenomic data have been submitted to the NCBI Sequence Read Archive (accession number: PRJNA1393893).

## Abbreviations

YCW: Yeast cell wall
ADG: Average daily gain
F/G: Feed to Gain
TVFA: Total volatile fatty acids
NH3-N: Ammonia nitrogen
G6PC: Glucose-6-phosphatase, catalytic subunit
PEPCK1: Phosphoenolpyruvate carboxykinase 1
FBP: Fructose-1,6-bisphosphatase
SREBP1c: Sterol regulatory element-binding protein 1c
AMPK: AMP-activated protein kinase
SCFAs: Short-chain fatty acids
RT-qPCR: Real-time fluorescent quantitative PCR
PCA: Principal component analysis
PCoA: Principal coordinate analysis
PLS-DA: Partial least squares discriminant analysis
KEGG: Kyoto Encyclopedia of Genes and Genomes
PC: Pyruvate carboxylase
ACC1: Acetyl-CoA carboxylase 1
FAS: Fatty acid synthase
CPT1: Carnitine palmitoyl transferase 1
PC: Phosphatidylcholine
PE: Phosphatidylethanolamine
SM: Sphingomyelin
ADP: Adenosine diphosphate
NAD+: Nicotinamide adenine dinucleotide
LPC: Lysophosphatidylcholine
LPA: Lysophosphatidic Acid
PPARα: Peroxisome proliferator-activated receptor alpha
TG: Triglyceride
T-CHO: Total Cholesterol
